# Detection of *Candidatus* Neoehrlichia mikurensis in Norway up to the northern limit of *Ixodes ricinus* distribution using a novel real time PCR test targeting the *groEL* gene

**DOI:** 10.1186/s12866-019-1502-y

**Published:** 2019-08-28

**Authors:** Andrew Jenkins, Cecilie Raasok, Benedikte N. Pedersen, Kristine Jensen, Åshild Andreassen, Arnulf Soleng, Kristin Skarsfjord Edgar, Heidi Heggen Lindstedt, Vivian Kjelland, Snorre Stuen, Dag Hvidsten, Bjørn-Erik Kristiansen

**Affiliations:** 1 Department of Natural Science and Environmental Health, University of South-Eastern Norway, Bø, Norway; 2 Present address: Nittedal Municipal Water and Drainage Authority, Nittedal, Norway; 3 Present address: Telemark Trust Hospital, Section for Pathology, Skien, Norway; 4 Department of Virology, Norwegian Institute of Public Health, Oslo, Norway; 5 Department of Pest Control, Norwegian Institute of Public Health, Oslo, Norway; 6 Department of Engineering and Science, University of Agder, Kristiansand, Norway; 7 Sørlandet Trust Hospital Research Unit, Kristiansand, Norway; 8 Department of Production Animal Clinical Sciences, Norwegian University of Life Sciences, Sandnes, Norway; 9 Department of Microbiology and Infection Control, University Hospital of North Norway, Tromsø, Norway; 10 Department of Process, Energy, and Environmental Technology, University of South-Eastern Norway, Porsgrunn, Norway

**Keywords:** *Neoehrlichia mikurensis*, *Ixodes ricinus*, Ticks, Tick-borne diseases, Norway, Scandinavia

## Abstract

**Background:**

*Candidatus* Neoehrlichia mikurensis is an emerging tick-borne pathogen. It is widely distributed in *Ixodes ricinus* ticks in Europe, but knowledge of its distribution in Norway, where *I. ricinu*s reaches its northern limit, is limited. In this study we have developed a real time PCR test for *Ca.* N. mikurensis and used it to investigate the distribution of *Ca.* N. mikurensis in Norway.

**Results:**

Real time PCR targeting the *groEL* gene was developed and shown to be highly sensitive. It was used to detect *Ca.* N. mikurensis in 1651 *I. ricinus* nymphs and adults collected from twelve locations in Norway, from the eastern Oslo Fjord in the south to near the Arctic Circle in the north. The overall prevalence was 6.5% and varied locally between 0 and 16%. Prevalence in adults and nymphs was similar, suggesting that ticks acquire *Ca.* N. mikurensis predominantly during their first blood meal. In addition, 123 larvae were investigated; *Ca.* N. mikurensis was not found in larvae, suggesting that transovarial transmission is rare or absent. Sequence analysis suggests that a single variant dominates in Norway.

**Conclusions:**

*Ca.* N. mikurensis is widespread and common in ticks in Norway and reaches up to their northern limit near the Arctic Circle. Ticks appear to acquire *Ca.* N. mikurensis during their first blood meal. No evidence for transovarial transmission was found.

## Introduction

*Candidatus* Neoehrlichia mikurensis (*Ca.* N. mikurensis) is an emerging tick-borne pathogen belonging to the order *Rickettsiales*, family *Anaplasmataceae*. Sequences corresponding to *Ca.* N. mikurensis were detected as early as 1999 in the Netherlands [1] and in 2001 in Norway [2] but their taxonomic position was not resolved; they were referred to informally as ‘*Ehrlichia*-like organism’ or ‘E. schotti’ although taxonomy of the genus *Ehrlichia* was at that time also unresolved.

The organism itself was first described in 2004 by Kawahara et al. [3] in rats (*Rattus norvegicus*) and *Ixodes ovatus* ticks from the Japanese island of Mikura. Sequence analyses showed that it was a new species within the recently reorganized [4] family *Anaplasmataceae* but that it did not belong to any of the existing genera. A new candidate genus, ‘Neoehrlichia’ was proposed to accommodate it and the name *Candidatus* Neoehrlichia mikurensis was proposed [3]. The first cases of human infection were reported from Sweden and Germany in 2010 [5, 6]. Neoehrlichiosis is primarily a disease of immunocompromised patients, who experience recurring fevers accompanied by a variety of other manifestations including musculoskeletal pain and deep-vein thrombosis [7]. Infections in immunocompetent persons may result in low-grade fever [8] or be asymptomatic [9]. Symptoms usually resolve quickly after treatment with tetracycline [7].

*Ca.* N. mikurensis appears to have a pan-Eurasian distribution, from Japan and China in the east [3, 8] to Spain in the west [10] and it has been found in all but one (Poland) of the 15 mainland European countries investigated so far [10–18]. In Western Europe, the tick host is *I. ricinus,* while in Russia it is *I. persulcatus* [19] and in Japan it is *I. ovatus* [3]. The main mammalian reservoir hosts for *Ca.* N. mikurensis appear to be wild rodents, including rats (*Rattus norvegicus*) [3], voles and mice [20–23]. Rodents are able to transmit *Ca.* N. mikurensis to xenodiagnostic ticks [22] and infection is widespread and common [17, 20–23]. There is also strong evidence for transplacental transmission in rodents [21]. Infections have also been detected in dogs [24] and hedgehogs [25], but not in shrews, moles or foxes [11, 19, 20, 23, 26].

Although *Ca.* N. mikurensis can be visualized by electron microscopy [3] and morulae may be detected in infected cells [9], the vast majority of studies have employed PCR-based methods. In earlier studies, detection was by 16S rDNA PCR followed by DNA hybridization [1, 2] or DNA sequencing [5] but more recently quantitative real time PCR (qPCR) tests targeting the 16S rDNA or *groEL* genes [11, 12, 27] have been applied. The latter methods are rapid, quantitative and less prone to contamination. Structural genes, such as *groEL*, have the advantage that they contain little secondary structure and it is easier to achieve specificity.

In this study, we describe the development and evaluation of a new real time PCR assay targeting the *groEL* gene of *Ca.* N. mikurensis and its use to determine the prevalence of *Ca.* N. mikurensis in *I. ricinus* ticks at localities throughout their northernmost habitat, the coastal regions of Norway, from the Oslo Fjord in the Southeast to the Arctic Circle in the North [28–31]. We also addressed the question of transovarial transmission of *Ca.* N. mikurensis by investigating a collection of *I. ricinus* larvae from a high-prevalence area.

## Materials and methods

### Tick collections and DNA extraction

Ticks were collected from vegetation by flag-dragging [32] or from dogs and cats brought to veterinary clinics [33]. DNA was extracted by (1) manual disruption and protease digestion [2], (2) mechanical disruption, automated total nucleic acid extraction and reverse transcription of total nucleic acid [33], (3) digestion with ammonium hydroxide [34, 35] or (4) phenol-chloroform extraction [36]. Table 1 describes the tick collections, the instar distribution and the method used for DNA extraction.

**Table 1:** Overview of tick collections

Location	Name^1^	Date (yyyy or yy-mm)	Larvae	Nymphs	Adults	Total	Source	Extraction method	Reference
1	Spjærøya (ØS)	12–09	–	67	–	67	Flagging	4	[37]
2	Håøya (AK)	13–05	–	95	–	95	Flagging	4	[37]
3	Brønnøya (AK)	13–06	–	92	–	92	Flagging	4	[37]
4	Langøya (TE)	00–04	63	–	–		Flagging	1	This work
		00–05	15	–	–		Flagging	1	This work
		00–06	25	–	–		Flagging	1	This work
		00–07	20	–	–		Flagging	1	This work
		All dates	123	–	–	123			
5	Langøya (TE)	00–05	–	47	25	72	Flagging	3	This work
		00–06	–	9	13	22	Flagging	3	This work
		01–05	–	1	26	27	Flagging	3	This work, [38]
		02–05	–	24	24	48	Flagging	3	This work, [38]
		03–05	–	25	–	25	Flagging	3	This work, [38]
		All dates		106	88	194			
6	Jomfruland (TE)	12–09	–	495	–	–	Flagging	1	[39]
7	Lower Telemark (TE)	2009	–	–	103	103	Dogs and cats	2	[33]
8	Tromøya (AA)	12–06	–	95	–	95	Flagging	4	[37]
9	Hillevågen (VA)	12–06	–	80	–	80	Flagging	4	[37]
10	Reme (VA)	00–07	–	48	51	99	Flagging	3	This work, [40]
11	Vindafjord (RO)	00–07	–	24	5	29	Flagging	3	This work, [40]
12	Stord/Borgundøy (HO)	00–07	–	26	47	73	Flagging	3	This work, [40]
13	Northern Norway (NO, TR)	2009	–	–	139	139	Dogs and cats	2	[33]

^1^Two-letter code in brackets indicates the county: ØS (Østfold); AK (Akershus); TE (Telemark); AA (Aust Agder); VA (Vest Agder); RO (Rogaland); HO (Hordaland); NO (Nordland); TR (Troms)

### Design of PCR

**Fig. 1:**
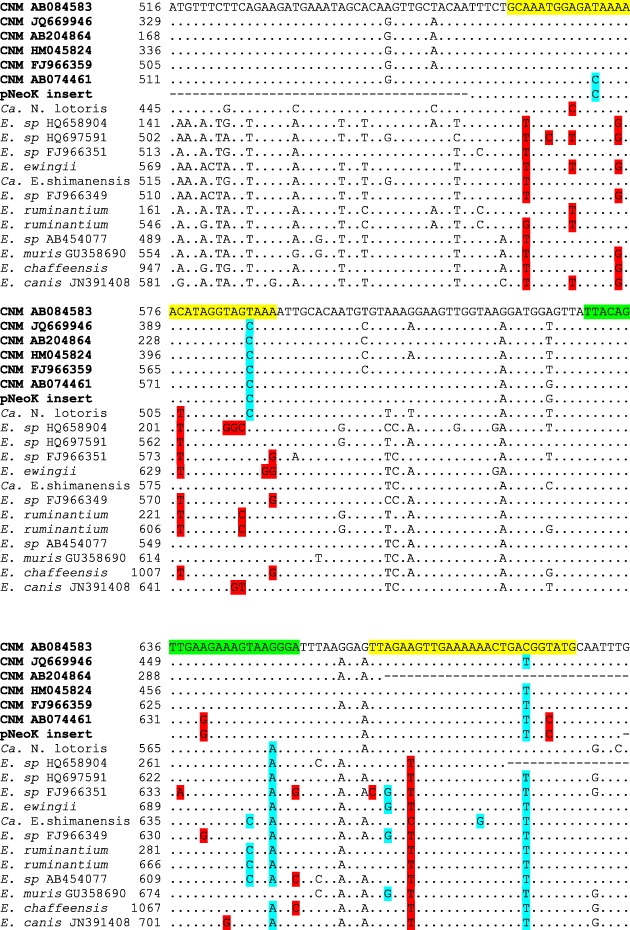
Multiple sequence alignment of the PCR target regions of *groEL* in *Ca.* N. mikurensis (CNM), *Ca.* N. lotori and selected *Ehrlichia* species. Dots indicate identity to the reference sequence, AB084583; letters indicate differences; hyphens indicate gaps or no sequence. The target regions for the primers and probe are highlighted in yellow and green respectively. Mismatches within the primer/probe target regions that give stable G:T basepairs are highlighted in blue. Destabilising mismatches (variants resulting in A:C, purine:purine or pyrimidine:pyrimidine) are highlighted in red. For reasons of space, sequence accession numbers for *Ca.* N. lotoris, *Ehrlichia ewingii*, *Candidatus* E. shimanensis, two sequence variants of *E. ruminantium* and *E. chafeensis* have been omitted from the figure; these are: EF633745, AF195273, AB074462, AB625796, DQ647005 and JQ085941 respectively

Our aim was to establish a real time PCR test that could be used either as a TaqMan PCR, with the extra specificity that the TaqMan probe potentially offers, or as a SYBR-green PCR, with the possibility of detecting sequence variants using standard single-derivative melting curves. A survey of *Ca.* N. mikurensis sequences available in April 2012 indicated that the *groEL* gene, which codes for a highly conserved heatshock protein [41], was a promising candidate for primer design. All available *Ca.* N. mikurensis *groEL *genes *per* 17.04.2012, together with *groEL* genes of *Candidatus* Neoehrlichia lotoris, *Ehrlichia muris*, *E. chafeensis, E. canis, E. ruminantium, E. ewingii,* *Ca.* E. shimanensis and unclassified *Ehrlichia* spp., were aligned using CLUSTALW; the alignments were displayed using BOXSHADE in order to identify sequence regions conserved within *Ca.* N. mikurensis but differing in other taxa. In order to select efficient primers, PrimerExpress v. 2.0 (Applied Biosystems, Foster City, CA, USA) was run, using *Ca.* N. mikurensis *GroEL* sequence AB084583 as the input sequence and program settings for design of TaqMan MGB™ real time PCR. The output primer and probe sequences were then compared with the multiple sequence alignment in order to identify sequences targeting suitable regions. This resulted in the selection of a probe and primers targeting the region 560–688 in AB084583. Figure 1 shows the sequence alignment and the positions of the primers and the probe. The primer and probe sequences were:

Forward Primer, Neo2f: GCAAATGGAGATAAAAACATAGGTAGTAAA.

Reverse Primer, Neo2r: CATACCGTCAGTTTTTTCAACTTCTAA.

Probe, Neo2m: TTACAGTTGAGGAAAGTAAGGGA (TaqMan MGB™ probe labelled with FAM (5(6)-carboxyfluorescein).

### Controls

In order to provide a quantifiable positive control, a synthetic plasmid, pNeo, was constructed according to our specifications (GenScript, Piscataway, NJ). pNeo is vector pUC57 with a de novo synthesized insert corresponding to positions 550–690 in sequence AB094461 (strain IS58). pNeo contains the 129 bp PCR target sequence plus 6 bp of flanking sequence on each side. AB094461 is the *Ca.* N. mikurensis *groEL* sequence with the greatest degree of divergence from the primer and probe sequences and was chosen in order to ensure a conservative estimate of PCR efficiency and sensitivity. Tenfold serial dilutions of pNeo from 1.6 × 10^9^ to 1.6 × 10^0^ copies per 5 μl aliquot were prepared and used in the determination of analytical sensitivity and PCR efficiency, optimization of primer and probe concentrations and standard curves for quantification of *Ca.* N. mikurensis in ticks. Alternatively, in some PCR runs, two tick samples containing an estimated 3 × 10^3^ and 6 × 10^4^ GU per reaction respectively were used as positive controls. These control samples were positive by the reverse line blot test and have been described in a previous study [2]. For evaluation of sensitivity and specificity, 38 tick samples from the latter study that had been analysed by the reverse line blot test were used. Samples for cross-reaction testing were four tick samples containing *Midichloria mitochondrii* (source: reference [2]), one tick sample containing *Wolbachia* (source: reference [2]), DNA from cultured *Ehrlichia chaffeensis* and *E. muris*, DNA from blood of an *E. canis* infected dog, DNA from blood of an *Anaplasma phagocytophilum* infected horse and DNA from blood of an *A. phagocytophilum* infected sheep. Control DNA was stored at − 20 °C when not in use; repeated freeze-thaw cycles were avoided.

### PCR

Real time PCR was run on the Applied Biosystems StepOne (Applied Biosystems, Foster City, CA, USA) using Applied Biosystems SYBR-green mastermix and primers Neo2f/Neo2r or TaqMan mastermix, the same primers, plus probe Neo2m as required. Primers were obtained from Applied Biosystems, Foster City, CA, USA or Integrated DNA Technologies, Leuven, Belgium. Except where otherwise stated, the SYBR-green PCR was used. The reaction volume was 25 μl, including 5 μl of template DNA, corresponding to 90 ± 60 ng for extraction methods 1 and 4 and 17 ± 10 ng for methods 2 and 3. Two positive controls (pNeo, 16 GU, 1.6 GU, or two positive tick samples containing an estimated 3 × 10^3^ and 6 × 10^4^ GU respectively), plus two negative controls (no DNA added) were included in each run of 48 samples. The PCR program was 50 °C, 2 min; 95 °C, 10 min, {95 °C, 15 s; 60 °C, 1 min} × 45 cycles. For runs using SYBR-green, dissociation analysis (60 °C to 95 °C with 0.3 °C increments) was appended to the program. Background subtraction, threshold setting, and the determination of Cq, Tm, and PCR efficiency were performed automatically by the instrument software (StepOne® and StepOnePlus®Real-Time PCR System Software Version 2.3); all instrument data was examined visually and manual corrections to threshold and background were made when necessary. Samples were considered positive if they displayed a detectable amplification curve rising above threshold on a logarithmic plot of fluorescence and a distinct melting peak at a temperature (Tm) between 71.7 °C and 75 °C. Where sufficient material was available, the TaqMan probe PCR was used to confirm positive results.

### DNA sequencing

The PCR products were purified using ExoSAP-IT (Applied Biosystems, Foster City, CA, USA) according to the manufacturer's instructions. BigDye Terminator v1.1 Cycle Sequencing Kit (Applied Biosystems, Foster City, CA, USA) was used to sequence PCR products directly in both forward and reverse direction, using primers Neo2r and Neo2f (sources as for PCR) on a 3130 Genetic Analyzer automated capillary sequencer (Applied Biosystems, Foster City, CA). The sequencing reactions were prepared and purified by ethanol precipitation according to the manufacturer’s instructions.

Forward and reverse sequences were assembled and edited using Chromas Pro v. 2.1.6 (Technelysium, Brisbane, Australia) and controlled by visual examination of the chromatograms. After trimming off the primer sequences, sequences were identified by BLAST search.

### Statistical methods

The 95% confidence intervals for prevalence estimates were calculated using the formulae:

$$ {P}_L=\frac{\left(2 np+{z}_{\alpha /2}^2-1\right)-{z}_{\alpha /2}\bullet \sqrt{z_{\alpha /2}^2-\left\{2+\left(1/n\right)\right\}+4p\left( nq+1\right)}}{2\left(n+{z}_{\alpha /2}^2\right)} $$

and

$$ {P}_U=\frac{\left(2 np+{z}_{\alpha /2}^2+1\right)+{z}_{\alpha /2}\bullet \sqrt{z_{\alpha /2}^2+\left\{2-\left(1/n\right)\right\}+4p\left( nq-1\right)}}{2\left(n+{z}_{\alpha /2}^2\right)} $$

for the lower and upper confidence limits respectively, where n is the number of samples, p and q are the proportions of positive and negative samples and z_α/2_ is the critical value of the normal distribution for α/2, in this case 1.96 [42]. The confidence limits are not valid if p or q ≤ 5/n; in such cases, no confidence interval was reported, except in the case of zero observed prevalence, where the upper 95% confidence limit may be calculated as ${P}_U=1-\sqrt[N]{0.05}$.

For significance testing the χ^2^ test was applied using Microsoft Excel.

## Results

### In silico assessment of the PCR test

Figure 1 shows that the primer and probe target sequences are somewhat variable among *Ca.* N. mikurensis strains, containing up to two mismatches to either primer or one mismatch to the probe. In all but one case, these are transition mutations that will allow the formation of a G:T base pair, which is nearly as stable as the canonical A:T. In one sequence, variants at the probe and reverse primer targets will result in destabilizing C:A mismatches. We deliberately chose this sequence as the insert in the positive control plasmid in order to provide the most stringent possible control of analytical sensitivity.

The most closely related species, *Ca.* Neoehrlichia lotoris and *Ehrlichia* spp., have at least two destabilizing mismatches in the forward primer target, together with smaller numbers of mismatches in the probe and reverse primer regions.

### Optimization of PCR

Forward and reverse primer concentrations of 100, 200, 400 and 800 nM were tested in all combinations. The effect of primer concentration on Cq values was slight. A primer concentration of 800 nM was chosen for all subsequent experiments. Probe concentrations of 50, 100, 200, 400, 800 and 1600 nM were tested; signal strength increased up to 200 nM; no improvement was found at higher probe concentrations.

### Comparison of SYBR-green and TaqMan modalities

**Fig. 2:**
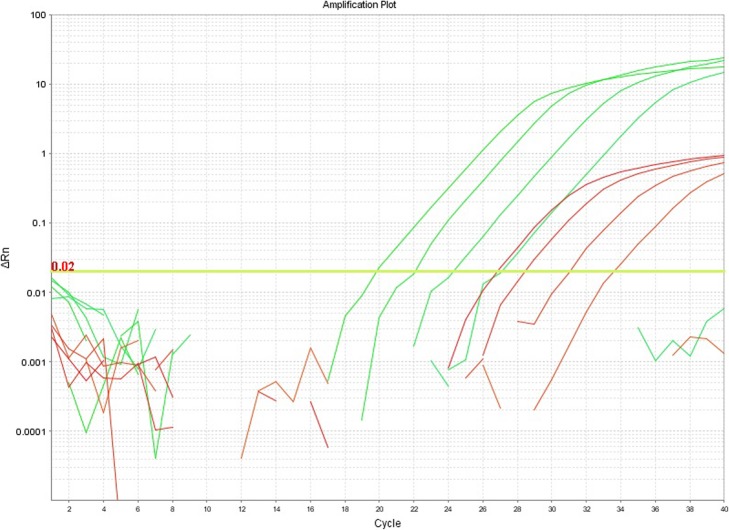
Comparison of TaqMan MGB Probe and SYBR green PCR. Amplification of a dilution series of an *I. ricinus* sample positive for *Ca.* N. mikurensis. Dilutions are 1:4, 1:20, 1:100, 1:500 and 1:2500 respectively. Green curves are for SYBR-green, red curves are for TaqMan MGB probe. The signals to the lower right of the amplification curves below the yellow-green threshold line are background noise from the 1:2500 dilutions

Figure 2 shows a comparison of amplification of a serial dilution of a positive control sample using SYBR-green and the TaqMan MGB probe respectively. While the detection limit (between sample dilutions of 1:500 and 1:2500) is the same for both modalities, SYBR-green gave Cq values that were 6.5–7 cycles lower and plateau signals that were approximately 20x higher, possibly as a result of intrinsic differences in fluorescence signal strength caused by fluorophor stoichiometry. As SYBR-green PCR gave stronger signals and the additional information of a Tm value for the amplicon, with the potential for detecting sequence variants, it was chosen for primary analysis of all samples.

### Efficiency and analytical sensitivity

**Fig. 3:**
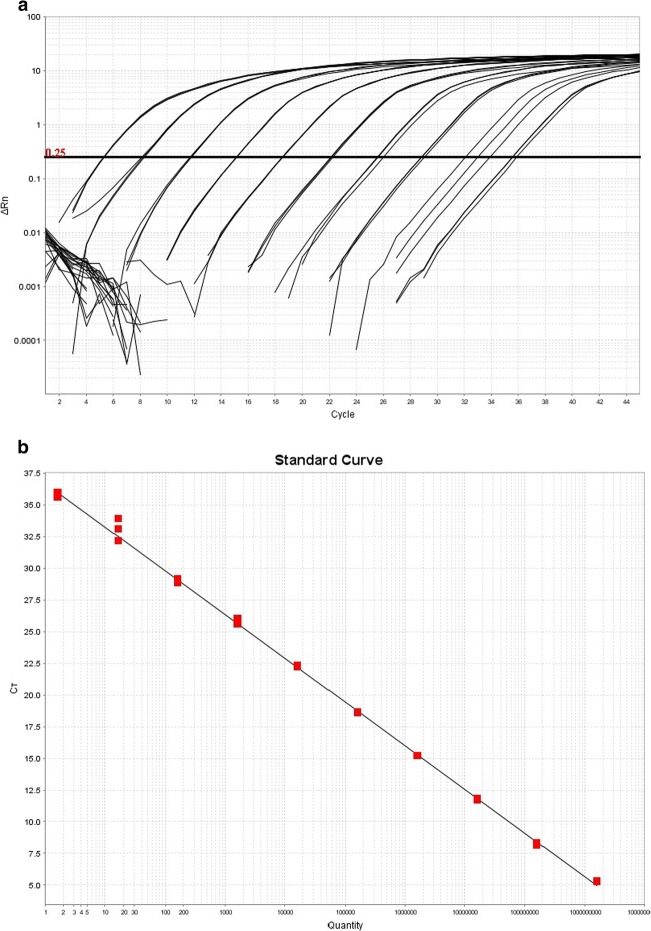
**a** Amplification curves for a 10x dilution series of pNeo containing from 1.6 × 10^9^ copies (leftmost curves) to 1.6 × 10^0^ copies (rightmost curves). **b** Standard curve of Cq values (CT) derived from (**a**) plotted against number of copies of the *groEL* gene (quantity; logarithmic scale)

PCR efficiency, estimated from a standard curve (Fig. 3) derived from a triplicate run of a dilution series of pNeo from 1.6 × 10^9^ to 1.6 copies/reaction, was 95%. The standard curve was linear (R^2^ = 0.999) throughout the range. All of three samples containing 1.6 copies/reaction were positive.

### Comparison with the reverse line-blot test

Thirty-eight ticks previously tested using the reverse line-blot test [1, 2] were tested with the current real time PCR test. Results are shown in Table 2. Relative to reverse line-blot, real time PCR showed 100% sensitivity. However, an additional four samples were positive with the real time PCR test. These four samples all showed very high Cq and/or anomalous Tm values.

**Table 2:** Comparison of the reverse line-blot (RLB) and *groEL* SYBR-green real time PCR methods for detection of *Ca.* N. mikurensis

		Real time PCR	
		Pos	Neg
RLB	Pos	12	0
	Neg	4	22

### Specificity

In order to test for cross-reaction with other members of the *Rickettsiales*, the current real time PCR test was run on samples containing *Anaplasma phagocytophilum* (*N* = 2), *Midichloria mitochondrii* (*N* = 4), *Wolbachia* (*N* = 1), *Ehrlichia canis* (*N* = 1), *Ehrlichia chaffeensis* (*N* = 1) and *Ehrlichia muris* (*N* = 1). Both *E. chaffeensis* and *E. muris* gave positive results; Tm was 73.9 for *E. chaffeensis* and 76.1 for *E. muris*; neither was positive with the TaqMan MGB probe. One of the four samples containing *M. mitochondrii* gave a very weak positive signal (Cq = 45) with a bimodal melting curve (Tm = 72.4, 74.9). All other samples were negative.

### Prevalence of *Ca.* N. Mikurensis in ticks

**Fig. 4:**
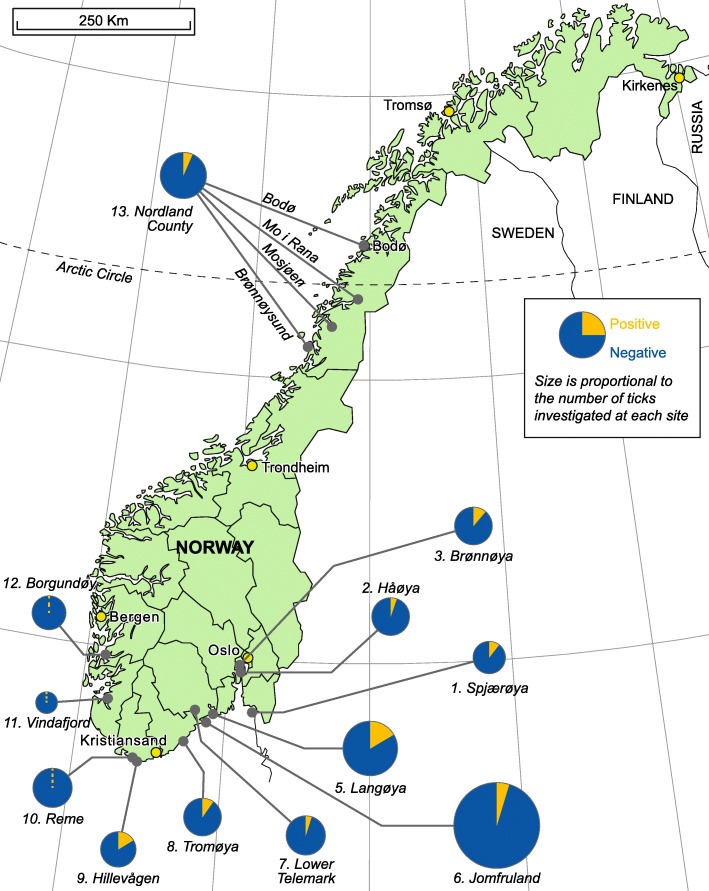
Map of Norway showing collection locations and the proportion of adult and nymphal ticks positive for *Ca.* N. mikurensis at each location. Location numbers correspond to location numbers in Table 5. The areas of the pie charts are proportional to the number of ticks. Collection 4 is not included as it includes only larvae. The locality is the same as collection 5

Table 3 and Fig. 4 show the prevalence of *Ca.* N. mikurensis in nymphal and adult ticks in the various collections based on SYBR-green PCR. The overall prevalence was 6.5%, and varied between zero and 16% at different localities. Cq values varied between 21 and 45; 3.7% of values were < 25; 89.8% were in the range 25–40 and 6.5% were > 40.

**Table 3:** Proportions of ticks positive for *Ca.* N. mikurensis

Collection	Location	Larvae	Nymphs	Males	Females	Total	% (CI)^a^
1	Spjærøy	–	7/67	–	–	7/67	10 (5–21)
2	Håøya	–	5/95	–	–	5/95	5 ()
3	Brønnøya	–	11/92	–	–	11/92	12 (6–21)
4	Langøya	0/123	–	–	–	0/123	0 (0–2.4)
5	Langøya	–	23/106	–	–	–	22 (15–31)
		–	–	7/48	–	–	15 (7–28)
		–	–	–	2/40	–	5 ()
						32/194	16 (12–23)
6	Jomfruland	–	23/495	–	–	23/495	5 (3–7)
7	Lower Telemark	–	–	–	5/103	5/103	5 ()
8	Tromøya	–	9/95	–	–	9/95	9 (5–18)
9	Hillevåg	–	13/80	–	–	13/80	16 (9–27)
10	Reme	–	0/48	0/25	0/26	0/101	0 (0–2.9)
11	Vindafjord	–	0/24	0/4	0/1	0/29	0 (0–10)
12	Stord	–	0/25	0/22	0/26	0/73	0 (0–4)
13	Nordland	–	–	–	9/139	9/139	6 (3–12)
All nymphs			79/1127	–	–	–	7 (6–9)
All males				7/99			7 (3–15)
All females					16/335		5 (3–8)
All adults						23/434	5 (3–8)
All ticks^b^						102/1561	6.5 (5.2-7.8)

^a^95% confidence interval in brackets. Where confidence intervals could not be calculated, this is indicated by empty brackets. ^b^Excluding larvae

All three tick collections with zero prevalence were collected in August 2000 at locations in the southern part of the western seaboard.

There was no significant difference in *Ca.* N. mikurensis prevalence between nymphs and adults or between males and females.

In order to investigate the possibility of PCR inhibition distorting our prevalence estimates, negative samples extracted using each of the four different extraction methods were spiked with 1000 GU of a *Ca.* N. mikurensis-positive sample and retested. Samples were considered partially inhibitory if they had an increased Cq value, completely inhibitory if no PCR signal was seen, and non-inhibitory if Cq was unchanged relative to a spiked aliquot of water. The results are shown in Table 4. The percentage of inhibitory samples was low with samples extracted with methods 1 and 3 (which includes the three zero-prevalence collections), while samples extracted with methods 2 and 4 had a high percentage of partial or complete inhibition. Thus, the prevalence estimates for collections 1, 2, 3, 7, 8, 9 and 13 must be considered lower bounds for the true prevalence.

**Table 4:** Spiking test for PCR inhibition in negative samples

Extraction method	N	Non-inhibitory	Partially inhibitory	Completely inhibitory
1	9	8	1	0
2	15	14	0	1
3	9	2	6	1
4	9	2	2	5

### Larvae

Larvae (*N* = 123) were analysed in pools of 5–9. None of the pools were positive. The same pools were then spiked with 1600 copies of pNeo and reanalysed. All pools were positive after spiking, indicating that these are true negative results and not the consequence of PCR inhibition. These larvae were collected in April – June 2000. In the previous year, the prevalence in female ticks at the same location was 8% [2].

### Tm variations, sequencing and confirmation by TaqMan MGB probe

SYBR-green PCR dissociation analysis gave amplicon Tm values varying between 71.7 and 74.5, with all but five samples in the range 72.2–74.2. Values for the plasmid control and the tick sample controls were 74.2 ± 0.5 and 73.4 ± 0.5 respectively. This, combined with the observed cross-reaction with *Ehrlichia* species suggested sequence variation in the amplicon and/or cross-reaction with other species. It was therefore considered desirable to confirm positive results using the TaqMan MGB probe Neo2m and/or by sequencing. Sixty-five SYBR-green positive samples were available for retesting. In all, 62/65 samples from eight locations were confirmed using the probe PCR, while 15/15 samples that generated readable sequence were confirmed as *Ca.* N. mikurensis by BLAST search (Table 5). All sequences were identical to the *Ca.* N. mikurensis sequence JQ669946. These samples had Tm values ranging from 72.2 to 74.1.

**Table 5:** Confirmation testing for ticks positive by SYBR-green PCR

Collection	Location	Total Pos (SYBR-green)	Confirmed by probe	Confirmed by sequencing
1	Spjærøy	7/67	NT	
2	Håøya	5/95	4/4	
3	Brønnøya	11/92	NT	
5	Langøya	32/194	18/18	3/3
6	Jomfruland	23/495	20/23	2/2
7	Lower Telemark	5/103	3/3	
8	Tromøya	9/95	1/1	
9	Hillevåg	13/80	11/11	7/7
10	Reme	0/101	NA	
11	Vindafjord	0/29	NA	
12	Stord	0/73	NA	
13	Nordland	9/139	5/5	3/3
All	–	125/1561	62/65	15/15

NA: not applicable; no ticks were positive. NT: not tested; no material was available for confirmation

## Discussion

In this study we have developed a new real time PCR test targeting the *groEL* gene of *Ca.* N. mikurensis. The test is highly sensitive, with samples containing as little as 1.6 DNA copies per 5 μl aliquot giving repeatably positive results. 12/12 samples in which *Ca.* N. mikurensis had previously been detected by reverse line-blot [1, 2] were also positive in the current PCR test, despite having been stored for more than 15 years.

The PCR may be run either with a TaqMan MGB™ probe or with SYBR-green combined with dissociation analysis. The analytical sensitivity is similar in either case. In this study we chose SYBR-green PCR as the primary test as it gave stronger signals and lower Cq values, and as we wished to investigate the possibility of using Tm analysis to detect sequence variants. A stronger signal is expected with SYBR-green, as each amplicon may bind multiple SYBR-green molecules, while for a TaqMan assay, only one probe fluorophore molecule is released from quenching per amplicon synthesized.

Although the amplicon Tm measured varied from 71.7 to 74.5 °C, this appears to be due to variations in experimental conditions or sample quality, as all amplicons sequenced (Tm range 72.2–74.1 °C) were identical to sequence JQ669946 and as large Tm deviations mostly disappeared after dilution of the sample (data not shown). The difference in Tm between sequences of the JQ669946 type present in samples (73.4 °C) and the AB094461 present in the positive control plasmid (74.2 °C) could be detected.

No cross-reaction with *Anaplasma phagocytophilum*, *Wolbachia* or *Ehrlichia canis* was observed. However, *E. chaffeensis* and *E. muris* gave significant cross-reaction. Both species have multiple mismatches to both primers, but these are well-removed from the 3' end. Although Fig. 2 indicates a destabilising mismatch at the 3′ end of the forward primer in *E. chaffeensis* (which would preclude amplification), this position is variable, with some sequences allowing formation of a canonical A:T base pair; we assume that it is such a variant that we have tested. A weak cross-reaction occurred with one of four samples containing *Midichloria mitochondrii*. A BLAST search of *M. mitochondrii groEL* sequences indicated eight mismatches with the forward primer and failed to detect any homology with the reverse primer sequence. This, and the fact that the other three samples were negative suggest that the apparent positive result was an experimental artefact rather than actual cross-reaction with *M. mitochondrii*. However, we cannot entirely exclude the possibility that the cross-reaction is to a different *M. mitochondrii* gene as yet unsequenced. Attempts to sequence the PCR product were not successful.

Our results indicate that the SYBR-green PCR reaction alone is too unspecific to definitively distinguish *Ca.* N. mikurensis from *Ehrlichia* species, or, by inference, other species of *Neoehrlichia*. This may in part be due to the high primer concentration used (800 nM); equally good results may be obtained with 300 nM and this is the concentration we now use. Tm analysis may help to constrain results, but we have found that this may vary by as much as 1.9 °C for identical sequences unless conditions are carefully controlled. Tm analysis would correctly flag *Ehrlichia muris* (Tm = 76.1) as a cross-reaction, but not *E. chaffeensis*, whose Tm (73.9) is within the normal range for *Ca.* N. mikurensis. However, these species are not known to occur in Northern Europe [1, 2].

In this study we have accepted the full range of observed Tm values (71.7–75) as positive, but our experience suggests that results at the extreme ends of this range (< 72.5 or > 74.5) should be regarded as potential cross-reactions or artefacts. Thus, confirmation of results with the TaqMan MGB probe and/or sequencing is desirable. In this study all of 15 samples sequenced and 62/65 samples tested with the probe were confirmed. This indicates that false positives represent only a minor component of our results and do not significantly bias our prevalence estimates. The three unconfirmed samples had high Cq values and we were unable to reproduce the original positive SYBR-green PCR result; it is possible that the amount of DNA remaining was not sufficient to generate a positive result.

Andersson et al. [12] have developed a real time PCR targeting another segment of the *groEL* gene of *Ca.* N. mikurensis. They found their PCR to be more sensitive than nested PCR targeting 16S rDNA and reported a prevalence in ticks in southern Sweden of 6%, which is close to that reported in this study. Vayssier-Taussat et al. [27] also describe a *groEL* real time PCR for *Ca.* N. mikurensis, although no sensitivity data are reported.

Our results extend knowledge of the prevalence of *Ca.* N. mikurensis to the northwestern limits of *I. ricinus* distribution and show that *Ca.* N. mikurensis is prevalent in *I. ricinus* throughout most of its range in Norway up to its northern limit, which is currently close to the Arctic Circle. A recent, more detailed study in the latter region confirms this [43]. Our results also confirm previous findings of *Ca.* N. mikurensis (then referred to as ‘*Ehrlichia*-like organism’) in ticks collected from southeastern Norway in 1999 [2]. Our findings are also consistent with the presence of *Ca.* N. mikurensis in ticks and wild rodents in neighboring Sweden [12, 20] and its apparently pan-European distribution [26]. The sequence variant found in this study matches variants that have been found in southern (JQ669946), eastern (KF312363) and northern Europe (LC167302) in mammals (KR912350), ticks (KF312363), and humans (EU810406) [9].

The overall prevalence in nymphs and adults was 6.5%. Where *Ca.* N. mikurensis was detected, the observed prevalence varied from location to location (5–16%). However, this study was designed to investigate the distribution of *Ca.* N. mikurensis in as many locations in Norway as possible using available material. As a prevalence study it has limitations: the nucleic acid extraction methods differed between locations; no attempt was made to control for failed extraction and there was evidence for PCR inhibition in more than half of the sample collections. For locations 7 and 13, the material used was reverse-transcribed total nucleic acid, a preparation chosen to allow detection of TBE-virus (an RNA virus) in the same material. As *groEL* is often strongly expressed, reverse transcription is expected to strengthen the PCR signal as both DNA and reverse-transcribed messenger RNA will be available for amplication. However, the mean Cq value for this material did not differ from that found for other methods (data not shown), possibly because the extraction volume was sixfold higher and the material thus represented a smaller proportion of the ticks’ DNA. False negative results and methodological biases are therefore possible and the local and overall prevalence estimates must be considered preliminary. Accurate prevalence estimates will require the use of standardised extraction methods and controls against inhibition and failed extraction.

In three locations no *Ca.* N. mikurensis was detected at all. These locations were all on or near the Western Seaboard and sampled in August 2000. PCR inhibition is not a major issue for these samples and nor is degradation, as *A. phagocytophilum* was successfully detected in the same samples in experiments run concurrently with this study (data not shown) using an *A. phagocytophilum*-specific real time PCR method [44] that uses the same PCR buffers and which amplifies a target of similar size to that in the present assay. Thus, we conclude that either *Ca.* N. mikurensis is locally scarce, or the ticks were collected at a point of time when *Ca.* N. mikurensis prevalence was low. Seasonal variations in *Ca.* N. mikurensis prevalence between 16% in May and 2% in June have been observed at location 5 (Fig. 1) [2]. This variation was paralleled by *Borrelia burgdorferi* sensu lato. Similar findings for *B. burgdorferi* sensu lato have been reported by Mysterud et al. [45].

*Ca.* N. mikurensis was not found in larvae (0/123), even though the larvae were collected in a high-prevalence area (location 5, Langøya) where *Ca.* N. mikurensis was detected in females the previous year. This result is in agreement with previous findings using real time PCR [11], and suggests that transovarial transmission of *Ca.* N. mikurensis is uncommon or absent, although conflicting results using nested PCR have been reported by Derdakova et al. [15] who found *Ca.* N. mikurensis in four of ten larvae.

In contrast to other tick-borne pathogens, such as TBE-virus [46] and *Borrelia* [47], the prevalence of *Ca.* N. mikurensis was not greater in adults than in nymphs. This suggests that *I. ricinus* ticks predominantly acquire *Ca.* N. mikurensis during their first blood meal and is consistent with small rodents, which are predominantly parasitized by larvae, being the main reservoir hosts for *Ca.* N. mikurensis [22, 26].

Our findings show that *Ca.* N. mikurensis is widespread in Norway, with a mean prevalence of 6.5%, making it the second most prevalent tick-borne pathogen after *Borrelia afzelii* [2, 37]. *Ca.* N. mikurensis has been recently detected in clinical specimens [48] and the first case of human neoehrlichiosis in Norway was recently reported [49].

## Data Availability

Data sharing is not applicable to this article as no datasets were generated or analysed during the current study.

## Abbreviations

Cq: Quantitation cycle. Fractional PCR cycle where an amplification curve crosses a threshold line. Also called Ct
GU: Genomic units. Number of copies of a DNA molecule corresponding to a single copy of the genome
MGB: Minor groove binder. A proprietary modification to TaqMan probes which increases Tm
Tm: Melting temperature of a DNA duplex
