# Correction to: Detection of Candidatus Neoehrlichia mikurensis in Norway up to the northern limit of Ixodes ricinus distribution using a novel real time PCR test targeting the groEL gene

**DOI:** 10.1186/s12866-020-1697-y

**Published:** 2020-01-10

**Authors:** Andrew Jenkins, Cecilie Raasok, Benedikte N. Pedersen, Kristine Jensen, Åshild Andreassen, Arnulf Soleng, Kristin Skarsfjord Edgar, Heidi Heggen Lindstedt, Vivian Kjelland, Snorre Stuen, Dag Hvidsten, Bjørn-Erik Kristiansen

**Affiliations:** 1 Department of Natural Science and Environmental Health, University of South-Eastern Norway, Bø, Norway; 2 Present address: Nittedal Municipal Water and Drainage Authority, Nittedal, Norway; 3 Present address: Telemark Trust Hospital, Section for Pathology, Skien, Norway; 4 Department of Virology, Norwegian Institute of Public Health, Oslo, Norway; 5 Department of Pest Control, Norwegian Institute of Public Health, Oslo, Norway; 6 Department of Engineering and Science, University of Agder, Kristiansand, Norway; 7 Sørlandet Trust Hospital Research Unit, Kristiansand, Norway; 8 Department of Production Animal Clinical Sciences, Norwegian University of Life Sciences, Sandnes, Norway; 9 Department of Microbiology and Infection Control, University Hospital of North Norway, Tromsø, Norway; 10 Department of Process, Energy, and Environmental Technology, University of South-Eastern Norway, Porsgrunn, Norway

**Correction to: BMC Microbiol**

**https://doi.org/10.1186/s12866-019-1502-y**

After publication of our article [1] it came to our notice that the source of the sequence for the control plasmid, pNeo (Materials and methods: Controls) was incorrectly stated as AB094461. The correct accession number is AB074461. The authors apologize for any confusion this may have caused.
